# MIR156-Targeted SPL9 Is Phosphorylated by SnRK2s and Interacts With ABI5 to Enhance ABA Responses in *Arabidopsis*

**DOI:** 10.3389/fpls.2021.708573

**Published:** 2021-07-21

**Authors:** Huixue Dong, Suli Yan, Yexing Jing, Ruizhen Yang, Yunwei Zhang, Yun Zhou, Yingfang Zhu, Jiaqiang Sun

**Affiliations:** ^1^National Key Facility for Crop Gene Resources and Genetic Improvement, Institute of Crop Sciences, Chinese Academy of Agricultural Sciences, Beijing, China; ^2^State Key Laboratory of Crop Stress Adaptation and Improvement, School of Life Sciences, Henan University, Kaifeng, China

**Keywords:** miR156, SPLs, ABA, ABI5, *Arabidopsis*

## Abstract

The miR156-targeted SQUAMOSA PROMOTER BINDING PROTEIN-LIKE (SPL) transcription factors play key roles in regulating plant development, but little is known about their function in abscisic acid (ABA) signaling. Here, we report that the miR156-targeted SPLs enhance ABA responses and contribute to the inhibition of pre-harvest sprouting. We find that SPL9 directly activates the expression of ABA responsive genes through binding to their promoters. SPL9 was further shown to physically interact with ABSCISIC ACID INSENSITIVE 5 (ABI5), a master transcription factor in ABA signaling, thus promoting its association with the promoters of ABA responsive genes. Furthermore, we reveal that the protein kinases SnRK2s interact with and phosphorylate SPL9, which is essential for its role in the activation of ABA responses. Together, our results disclose a SnRK2s-SPLs-ABI5 regulatory module in ABA signaling in *Arabidopsis*.

## Introduction

The stress-related phytohormone abscisic acid (ABA) inhibits seed germination and seedling growth to adapt various environmental challenges (Cutler et al., 2010; Weiner et al., 2010). Molecular genetics studies have significantly advanced our understanding on the molecular basis of ABA signaling in *Arabidopsis*. Among them, ABA-INSENSITIVE1 (ABI1) (Leung et al., 1994; Gosti et al., 1999) and ABI2 (Leung et al., 1997; Rodriguez et al., 1998) are clade A protein phosphatase 2Cs (PP2Cs), which negatively regulate ABA signaling during seed germination. However, the downstream B3 transcription factor ABI3, AP2 transcription factor ABI4 and bZIP transcription factor ABI5 positively regulate the ABA-inhibited seed germination and early seedling development (Giraudat et al., 1992; Finkelstein et al., 1998, 2011; Finkelstein and Lynch, 2000; Umezawa et al., 2010). Several SNF1 (sucrose non-fermenting 1)-related kinase 2s (SnRK2s), including SnRK2.2, SnRK2.3, and SnRK2.6 (also known as Open Stomata 1, OST1), were identified as stress- or ABA-activated protein kinases and function redundantly in ABA-mediated regulation of seed germination, seedling growth, drought stress and stomatal closure (Mustilli et al., 2002; Fujii et al., 2007; Nakashima et al., 2009; Ding et al., 2015; Huang et al., 2018).

Since the identification of ABA receptors, PYRABACTIN RESISTANCE1 (PYR1)/PYR1-LIKE (PYL)/REGULATORY COMPONENTS OF ABA RECEPTORS (RCAR) (Fujii et al., 2009; Melcher et al., 2009; Miyazono et al., 2009; Santiago et al., 2009; Soon et al., 2012), a core ABA signaling pathway has been discovered. In the absence of ABA, PP2Cs inhibit the activity of SnRK2s by physical interaction and dephosphorylation (Fujii et al., 2009; Ma et al., 2009; Umezawa et al., 2009), leading to inhibition of downstream transcription factors required for ABA-responsive gene expression (Kobayashi et al., 2005). Perception of ABA by its receptors PYR/PYL/RCAR, facilitates the interaction between PYR/PYL/RCAR and PP2Cs to prevent PP2Cs inhibition on SnRK2s activity (Fujii et al., 2009; Ma et al., 2009; Park et al., 2009). Thus, the ABA-activated SnRK2s phosphorylate and activate the downstream transcription factors (e.g., ABI5) to regulate ABA responsive gene expression (Kobayashi et al., 2005; Fujii et al., 2007; Nakashima et al., 2009).

The SQUAMOSA PROMOTER BINDING PROTEIN (SBP)-like (SPL) belongs to plant-specific transcription factors and contains a highly conserved SBP-box domain (Cardon et al., 1999), which was revealed to specifically bind the core cis-element GTAC (Yamasaki et al., 2004; Birkenbihl et al., 2005; Liang et al., 2008; Lu et al., 2013). In *Arabidopsis*, *SPL* genes are divided into two subgroups, represented by *SPL3* (including *SPL3*, *SPL4*, and *SPL5*) which encodes a small protein, and *SPL9* (including *SPL2*, *SPL6*, *SPL9*, *SPL10*, *SPL11*, *SPL13*, and *SPL15*) which encodes a much larger protein, respectively (Cardon et al., 1999; Yang et al., 2008). Among them, some *SPL* genes such as *SPL3*, *SPL9*, and *SPL15* are regulated by microRNA156 (miR156) (Schwab et al., 2005; Wu and Poethig, 2006; Xing et al., 2010). The miR156-targeted SPL transcription factors play key roles in plant growth and development. For example, *SPL3*, *SPL4*, *SPL5*, and *SPL9* function in the control of flowering time and phase transition (Wu and Poethig, 2006; Wang et al., 2009); *SPL9* and its paralog *SPL15* regulate shoot branching (Schwarz et al., 2008). In addition, recent studies reported that overexpression or knockdown of miR156 can affect seed germination and dormancy in *Arabidopsis* and rice (Huo et al., 2016; Liu et al., 2019; Miao et al., 2019).

In this study, we uncover that miR156-targeted SPLs transcription factors positively regulate ABA responses and inhibit pre-harvest sprouting (PHS) in *Arabidopsis*. We demonstrate that SPLs interact with the master transcription factor ABI5 to promote ABA signaling. Furthermore, we show that SnRK2s physically interact with and phosphorylate SPLs. Importantly, the ABA-induced SPL9 phosphorylation is required for its function in the activation of ABA responses.

## Materials and Methods

### Plant Materials and Growth Conditions

*Arabidopsis thaliana* ecotype Col-0 was used as the wild type. Some of the plant materials used in this study were previously described: *GFP-rSPL9* (Wang et al., 2009); *rSPL3-HA* (Wang et al., 2009); *MIM156* (Wang et al., 2009); *MIR156* (Xie et al., 2017); *abi5-7* (Chen et al., 2012), and *snrk2.2/2.3/2.6* (Fujii and Zhu, 2009). The *GFP-rSPL9/abi5-7* and *GFP-rSPL9/snrk2.2/2.3/2.6* lines were generated by genetic crossing between *GFP-rSPL9* and *abi5-7* or *snrk2.2/2.3/2.6*, respectively.

*Arabidopsis* seedlings were grown on half-strength Murashige and Skoog (MS) solid medium containing 2% sucrose at 22°C in a light incubator with 16-h-light/8-h-dark photoperiod. *N. benthamiana* plants were grown under a 16-h-light/8-h-dark cycle in a greenhouse at 22°C for 1 month before infiltration.

### DNA Constructs and Transgenic Plants

For BiFC assays, gateway cloning strategy (Invitrogen) was used. The full-length coding sequence (CDS) of *SPL9* or *SPL3* was cloned into *pQBV3* vector (Dong et al., 2020) and subsequently introduced into the destination vector *pEarleygate202-YN* (cYFP) (Lu et al., 2010). Similarly, the full-length CDS of *ABI5* was introduced into the *pEarleygate201-YN* (nYFP) vector (Lu et al., 2010).

For LCI assays, the full-length CDS of *SPL9* was cloned into *p1300-35S-nLUC* vector or *p1300-35S-cLUC* vector (Chen et al., 2008) to generate nLUC-SPL9 or cLUC-SPL9. Similarly, the CDSs of *ABI3*, *ABI4*, *ABI5*, and *SnRK2s* were cloned into *p1300-35S-nLUC* vector or *p1300-35S-cLUC* vector (Chen et al., 2008), respectively. The truncated versions of *SPL9* or *ABI5* were cloned into *p1300-35S-cLUC* vector (Chen et al., 2008), respectively.

For pull-down assays, the full-length CDS of *SPL9* or *SPL3* was inserted into *pMAL-c2X* vector to generate MBP-SPL9 and MBP-SPL3, respectively. Then, the MBP-SPL9 construct was mutated to MBP-SPL9(2A) using the Site-Directed Mutagenesis Kit (Mei5 Biotechnology, MF129-01). Similarly, the full-length CDS of *ABI5* was inserted into *pGEX4T-1* vector to generate ABI5-GST. All the ligations above were performed based on ligation free cloning master mix (Applied Biological Materials, E011-5-A) according to the manufacturer’s instruction.

For *Em6_pro_:LUC* and *Em1_pro_:LUC* constructs, the ∼1.5-kb promoter of *Em6* and 800-bp promoter of *Em1* were separately ligated into the entry vector *pQBV3*, and then introduced into the vector *pGWB35* (Nakagawa et al., 2007). The construct of *35S:rSPL9-MYC* was generated based on the destination vector *pGWB17* (Nakagawa et al., 2007). The constructs of *rSPL9-YFP* and *rSPL9(2A)-YFP* were generated based on the destination vector *pEarly-101* driven by the 35S promoter.

To generate the *SPL9_pro_:GFP-rSPL9*, *SPL9_pro_:GFP-rSPL9(2A)* and *GFP-rSPL9/SnRK2.6-Flag* transgenic plants, 2-kb promoter of *SPL9* was ligated into *p1305-35S-GFP* to produce *p1305-SPL9_pro_-GFP*, next the full length CDS of *SPL9* or *SPL9(2A)* was introduced in it to generate *SPL9_pro_:GFP-rSPL9* or *SPL9_pro_:GFP-rSPL9(2A)* construct, respectively. *SnRK2.6* gene was amplified and inserted into the *p1300-35S-Flag* vector. *Agrobacterium* strain GV3101 carrying the construct was then transformed into the Col-0 or *GFP-rSPL9* plants to generate *SPL9_pro_:GFP-rSPL9*, *SPL9_pro_:GFP-rSPL9(2A)* or *GFP-rSPL9/SnRK2.6-Flag* transgenic plants using the floral-dip method (Clough and Bent, 1998), respectively.

All the primers used for the constructs above are summarized in Supplementary Table 1 and the constructs described above are summarized in Supplementary Table 2.

### RNA Extraction and Gene Expression Analyses

Total RNA was extracted using Trizol (Invitrogen) reagent according to the manufacture’s instruction. About 2 μg of total RNA were used for reverse transcription with the 5× All-In One RT MasterMix system (Applied Biological Materials). Quantitative real-time polymerase chain reaction (qRT-PCR) assay was performed using SYBR^®^ Premix Ex Taq Kit (TaKaRa), and the expression levels of *ACT7* were used as the internal control. The primer sequences are listed in Supplementary Table 3.

### ABA Treatment Assays and Pre-Harvest Sprouting

For ABA responses, seeds of different genotypes were harvested at the same time for the germination and cotyledon greening assays as described before (Bu et al., 2009; Li et al., 2011). Seeds of different genotypes were sown on the same 1/2 MS medium supplemented with different ABA concentrations as indicated and chilled at 4°C in the dark for 2 days (stratified). Then the seeds were moved to 22°C with a 16-h-light/8-h-dark cycle in a light chamber. The percentage of seed germination or cotyledon greening was scored at 3 or 5 days after the end of stratification, respectively. Germination was defined as an obvious emergence of the radicle through the seed coat. Cotyledon greening is defined as obvious cotyledon expansion and turning green (Bu et al., 2009; Li et al., 2011; Chen et al., 2012). For the PHS test, plants with early siliques that matured at the same time were directly sown on water saturated filter paper then placed in the growth chamber with a 16-h-light/8-h-dark cycle.

### Firefly Luciferase Complementation Imaging (LCI) Assays

The luciferase complementation imaging (LCI) assays for the protein interaction detection was performed in *N. benthamiana* leaves as described previously (Chen et al., 2008). The indicated genes were fused into nLUC or cLUC, respectively, and separately introduced into *Agrobacterium* strain GV3101. Then, *Agrobacteria* cells carrying nLUC or cLUC derivative constructs were co-injected in *N. benthamiana* leaves. The LUC activities were analyzed using NightSHADE LB 985 (Berthold).

### Chromatin Immunoprecipitation-qPCR Assays

The 6-day-old *Arabidopsis* seedlings grown on 1/2 MS medium were treated with or without 50 μM ABA for 2 h and then collected for chromatin immunoprecipitation (ChIP) assays as previously described (Zhu et al., 2012). Briefly, about 2 to 3 grams of each sample were cross-linked in 1% formaldehyde under vacuum for 15 min, followed by 5-min neutralization with 0.125 M glycine. The samples were separately immunoprecipitated with or without anti-GFP antibody (Abcam, ab290). Finally, the GFP-specific enrichment of the fragments from *Em1* or *Em6* promoter was analyzed by qPCR using specific primer sets listed in Supplementary Table 4. The enrichment fold of a certain fragment was calculated by normalizing to the amount of no antibody-immunoprecipitates DNA samples.

### Subcellular Localization and Bimolecular Fluorescence Complementation (BiFC) Assays

For localization experiments, *Agrobacterium* GV3101 harboring the *rSPL9-YFP* or *rSPL9(2A)-YFP* construct was injected into *N. benthamiana* leaves. For BiFC assays, the indicated vectors were co-transformed into *Agrobacterium* GV3101 and then co-expressed in *N. benthamiana* leaves as described previously (Dong et al., 2020). The injected tobacco leaves were incubated for 48 h, and then the fluorescence signal of yellow fluorescent protein (YFP) was observed using the confocal microscope (Carl Zeiss, LSM880).

### Protein Extraction, Immunoblotting, and Co-immunoprecipitation (Co-IP) Analyses

The GFP-SPL9 fusion proteins were extracted from the 6-day-old *GFP-rSPL9* or *GFP-rSPL9(2A)* transgenic plants using the extracted buffer (125 mM Tris–HCl [pH 6.8], 4% SDS, 20% glycerol, 0.001% Bromophenol blue, 2% β–Mercaptoethanol). For the immunoblotting detection of GFP-SPL9, we used anti-GFP (1:2000; Roche, 11814460001) antibody. ACT (1:5000; CWBIO, CW0264) was employed as a loading control.

The Col-0, *GFP-rSPL9* transgenic plants and anti-ABI5 antibody were used in the Co-IP assays for the interaction of SPL9 and ABI5. Total proteins were extracted from the 6-day-old seedlings treated with 50 μM ABA for 2 h using the lysis buffer (50 mM Tris–HCl [pH 7.5], 150 mM NaCl, 5 mM EDTA [pH 8.0], 0.2% Triton X-100, 0.2% NP-40, 20 μM MG132) with freshly added PMSF (0.6 mM) and 1× protease inhibitor. The extracts were centrifuged for 20 min and the supernatant was incubated with anti-GFP magnetic beads (MBL, D153-10) overnight. Next, the beads were washed five times with the lysis buffer and eluted samples were analyzed by immunoblotting with anti-GFP and anti-ABI5 (1:5000; Agrisera, AS121863) antibodies.

The 6-day-old *GFP-rSPL9* and *GFP-rSPL9/SnRK2.6-Flag* transgenic plants treated with 50 μM ABA plus 30 μM MG132 for 4 h were used in the Co-IP assays for the interaction of SnRK2.6 and SPL9. Total proteins were extracted as described above. The supernatant was incubated with anti-Flag magnetic beads (MBL, M185-10) overnight. Proteins were detected with anti-GFP and anti-Flag (1:5000; MBL, M185-3L) antibodies, respectively.

### *In vitro* and Semi-*in vivo* Pull-Down Assays

The constructs (MBP, MBP-SPL9, MBP-SPL3, GST, and ABI5-GST) were separately transformed into *Escherichia coli* transetta. The fusion proteins were induced with 4 mM isopropyl β-D-thiogalactopyranoside (IPTG) at 18°C overnight. For the pull-down assays of SPL9 and ABI5, the fusion proteins were incubated with glutathione resin (GenScript) overnight in column buffer (20 mM Tris–HCl [pH 7.5], 200 mM NaCl, 1 mM PMSF, 1 mM DTT and 1× protease inhibitor (Roche 4693132001)]. For the pull-down assays of SPL3 and ABI5, the fusion proteins were incubated with amylose resin (New England Biolabs) overnight in column buffer. Next, the GST bind resin or MBP bind resin was washed five times with column buffer, resolved by SDS-PAGE, and detected using anti-GST (1:3000, CW0144, CWbiotech) and anti-MBP (1:3000, CW0288, CWbiotech) antibodies.

The 6-day-old *GFP-rSPL9* seedlings treated with 50 μM ABA for 2 h and SnRK2.6-His fusion proteins were used for the semi-*in vivo* pull-down assays. The GFP-SPL9 proteins were extracted with lysis buffer with freshly added PMSF (0.6 mM) and 1× protease inhibitor. Then SnRK2.6-His fusion proteins were incubated with the GFP-SPL9 protein extracts overnight and added Ni-NTA resin (TransGen Biotech, DP101-01) for a further 2 h. The His bind resin was washed five times with PBS buffer (CWBIO, CW0040S), resolved by SDS-PAGE, and detected using anti-His (1:3000; CWBIO, CW0143M) and anti-GFP (1:2000; Roche, 11814460001) antibodies, respectively.

### *In vitro* and *in vivo* Phosphorylation Assays

For the *in vitro* phosphorylation assays, 1 μg MBP-SPL9, MBP-SPL9(2A) or MBP-SPL3 fusion proteins were incubated with 1 μg SnRK2.6-His in 20 μl kinase reaction buffer (25 mM Tris–HCl [pH 7.5], 12 mM MgCl_2_, 1 mM DTT and 1 mM ATP) at 37°C for 1 h. The reactions were boiled with 5× SDS loading buffer then separated by phos-tag SDS-PAGE (Kinoshita et al., 2006). The signals were detected using anti-MBP antibody.

For the *in vivo* kinase assays, the GFP-SPL9 fusion proteins were extracted with buffer (150 mM KCl, 50 mM HEPES [PH7.5], 0.4% Triton X-100, 1 mM DTT, 1× protease inhibitor and phosphatase inhibitor cocktail) and immunoprecipitated with anti-GFP magnetic beads. Then the IP products were separated by phos-tag SDS-PAGE and analyzed with anti-GFP antibody.

### Transcriptional Activity Assays in *N. benthamiana*

The transcriptional activity assays were carried out in *N. benthamiana* leaves as previously described (Sun et al., 2012). In brief, the reporter *Em_pro_:LUC* and effector *35S:rSPL9-MYC* were separately introduced into *Agrobacterium* GV3101 to perform the con-infiltration in *N. benthamiana* leaves. The *N. benthamiana* leaves after infiltrating 24 h were injected with 50 μM ABA and incubated for a further 24 h. The luciferase luminescence was observed using NightSHADE LB 985 (Berthold), and quantification of luciferase activities were carried out with IndiGO software (version 2.03.0).

### Accession Numbers

Sequence data from this article can be found in the Arabidopsis Genome Initiative or GenBank/EMBL databases under the following accession numbers: *SPL9* (At2g42200), *SPL3* (At2g33810), *ABI5* (At2g36270), *ABI3* (At3g24650), *ABI4* (At2g40220), *SnRK2.2* (AT3G50500), *SnRK2.3* (AT5G66880), *SnRK2.6* (AT4G33950), *Em1* (AT3G51810), and *Em6* (AT2G40170).

## Results

### The miR156-Targeted SPLs Enhance ABA Responses

**FIGURE 1:**
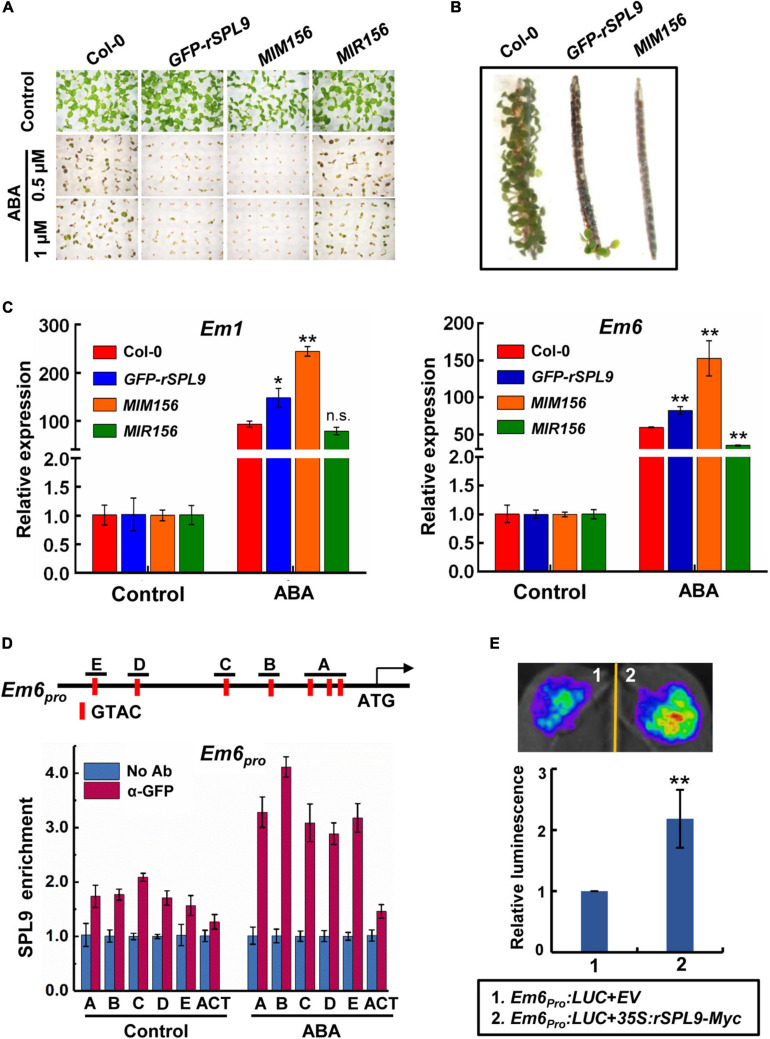
The miR156-targeted SPLs enhance ABA responses. **(A)** Germination phenotype of the indicated seedlings grown on medium containing 0, 0.5, or 1 μM ABA for 7 days. **(B)** Pre-Harvest Sprouting phenotype of the indicated genotypes in fresh mature siliques. **(C)** qRT-PCR assays showing the expression patterns of ABA-responsive genes in 4-day-old seedlings of indicated genotypes with 10 μM ABA treatment (4 h ABA treatment for *Em1*; 2 h ABA treatment for *Em6*). The expression levels in untreated seedlings (Control) for each genotype were set to one. Data are means ± SD (*n* = 3). Asterisks indicate significant differences between the Col-0 and transgenic seedlings. *P < 0.05, **P < 0.01, n.s. indicates no significant difference (Student *t*-test). **(D)** Chromatin immunoprecipitation (ChIP)-qPCR assays showing the enrichment of SPL9 at the *Em6* promoter regions. The vertical red lines in the upper panel indicate the positions of SBP-box binding core motifs. The 6-day-old *GFP-rSPL9* seedlings treated without (Control) or with 50 μM ABA for 2 h were harvested for ChIP assays. Error bars denote ± SD (*n* = 3). *ACT7* was used as a control. **(E)** Transient expression assays illustrating the activation of *Em6* promoter by SPL9. Upper panel shows a representative leaf image, and the column diagram represents relative luminescence intensities (*n* = 15). The mean value in combination one was set to one. **P < 0.01, (Student’s *t*-test).

To investigate a potential role of the miR156-regulated SPLs in the ABA signaling, we tested the seed germination and cotyledon greening phenotypes of SPLs-related transgenic lines in response to ABA. The *GFP-rSPL9* line is identical to a gain-of-function mutant of *SPL9* gene, in which a miR156-resistant version of *SPL9* is expressed from its native promoter (Wang et al., 2009), and the *MIM156* line has elevated expression of *SPL9* and other *SPLs* (Wang et al., 2009). In the absence of exogenously supplied ABA, the seed germination and cotyledon greening percentages of different genotypes were comparable (Figure 1A and Supplementary Figure 1). However, the seed germination and cotyledon greening of *GFP-rSPL9* and *MIM156* seedlings were much lower than the wild-type Columbia-0 (Col-0) under ABA treatment (Figure 1A and Supplementary Figure 1), indicating that overexpression of SPL9 and SPL3 conferred ABA hypersensitivity. Thus, the miR156-regulated SPL9 appears to play a positive role in regulating ABA responses. Meanwhile, SPL3 also positively regulates ABA responses in seed germination and cotyledon greening (Supplementary Figure 1). These results suggest that the miR156-targeted SPLs play an enhancing effect on the ABA response during seed germination and early seedling development.

Considering that ABA plays a critical role in preventing PHS, which occurs when adequate temperature and humid conditions prevail during late maturation of crops in the field, we wondered whether the miR156-SPL9 module plays a role in preventing PHS. We conducted germination assays using mature siliques of the Col-0, *GFP-rSPL9* and *MIM156* plants. Interestingly, the freshly harvested seeds from unopened siliques of *GFP-rSPL9* and *MIM156* displayed greatly increased dormancy compared with Col-0 (Figure 1B).

We further investigated whether the miR156-targeted SPLs regulate the transcriptional expression of ABA responsive genes. Quantitative reverse transcriptase-PCR (qRT-PCR) analyses showed that ABA-induced expression levels of the representative ABA-responsive genes *Em1* and *Em6* were dramatically enhanced in the *GFP-rSPL9* and *MIM156* seedlings compared with Col-0 plants (Figure 1C). In contrast, the ABA-induced expression levels of *Em6* in *MIR156* seedlings were obviously lower than those in WT plants (Figure 1C), demonstrating again that the miR156-targeted SPLs enhance ABA responses.

### SPL9 Directly Activates the Expression of ABA-Responsive Genes

The above findings that SPLs enhance ABA responses promoted us to study whether SPLs directly bind to the promoters of ABA-responsive genes. As plant-specific transcription factors, SPLs predominantly bind to the common SBP-binding motifs (such as GTAC) of target genes (Birkenbihl et al., 2005; Liang et al., 2008; Lu et al., 2013). We first scanned the *Em6* (∼1.5-kb) promoter sequence and identified seven putative SBP-binding motifs with positions of −197/−200, −229/−232, −285/−288 (labeled as A), −427/−430 (labeled as B), −626/−629 (labeled as C), −1043/−1046 (labeled as D), and −1220/−1223 (labeled as E), respectively, (Figure 1D). Next, we performed chromatin immunoprecipitation-quantitative PCR (ChIP-qPCR) assays using the 6-day-old *GFP-rSPL9* seedlings treated without (control) or with 50 μM ABA for 2 h. The results showed that the enrichment of SPL9 at *Em6* promoter was relatively low in the absence of ABA, whereas ABA treatment substantially increased the enrichment of SPL9 at the *Em6* promoter (Figure 1D). Similarly, we found two SBP-binding motifs in the *Em1* promoter (800-bp) with positions of −382/−385 (labeled as A) and −675/−678 (labeled as B) (Supplementary Figure 2A). The ChIP-qPCR assays showed that SPL9 was also deposited in the *Em1* promoter, especially when treated with ABA (Supplementary Figure 2A), implying that ABA can stimulate the deposition of SPL9 to the promoters of ABA-responsive genes.

We further performed transient transcriptional activation assays in *Nicotiana benthamiana* to determine the effect of SPL9 on the transcription of target genes. The *Agrobacterium* strains harboring different constructs, including the *Em6_pro_:LUC* reporter and the effector *35S:rSPL9-Myc*, were co-infiltrated into *N. benthamiana* leaves. The results showed that transient expression of SPL9 could intensely elevate the expression of *Em6_pro_:LUC* reporter (Figure 1E). Similarly, the luminescence intensities of *Em1_pro_:LUC* were significantly enhanced when co-expressing with *35S:rSPL9-Myc* (Supplementary Figure 2B). These results further suggest that SPL9 could directly activate the transcription of ABA-responsive genes.

### SPLs Physically Interact With ABI5

**FIGURE 2:**
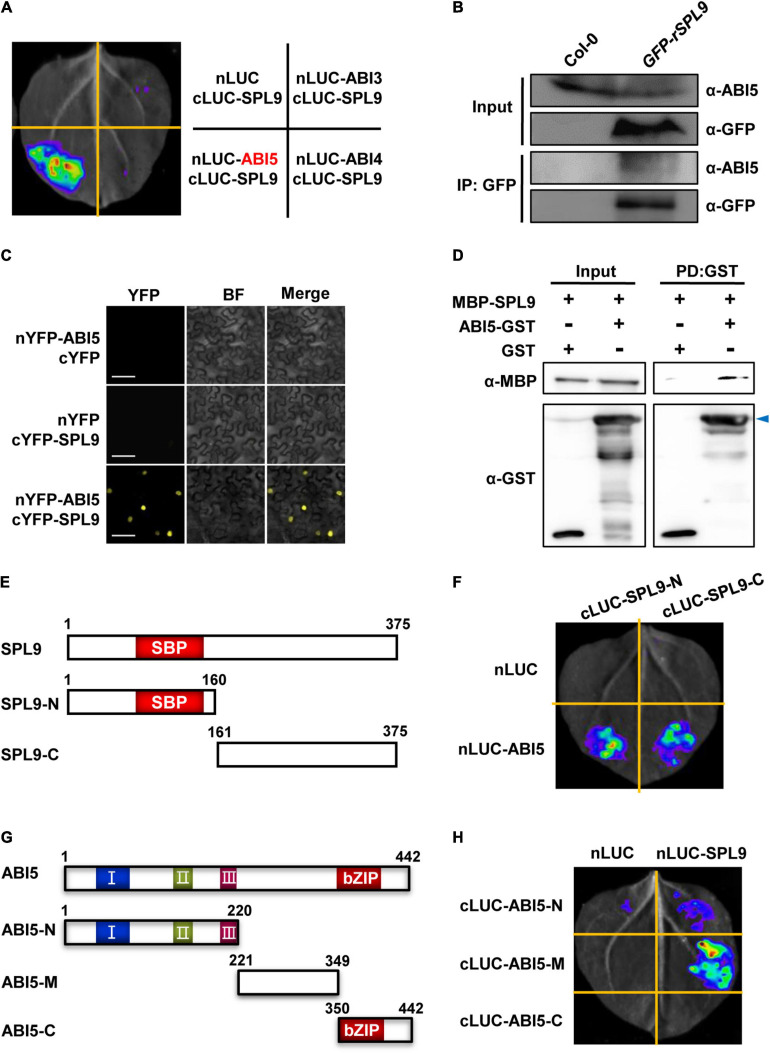
SPL9 physically interacts with ABI5 *in vitro* and *in vivo*. **(A)** Luciferase complementation imaging (LCI) assays showing that SPL9 interacts with ABI5. The cLUC-SPL9 and cLUC-ABI3/ABI4/ABI5 were co-transformed into *Nicotiana benthamiana* leaves, respectively. **(B)** Co-immunoprecipitation (Co-IP) assays showing that SPL9 physically interacts with ABI5 *in vivo*. Total proteins were extracted from the 6-day-old seedlings treated with 50 μM ABA for 2 h. The immunoprecipitates were detected using anti-ABI5 and anti-GFP antibodies, respectively. **(C)** Bimolecular fluorescence complementation (BiFC) assays showing the interaction of SPL9 and ABI5. The constructs indicated were co-transformed into *N. benthamiana* leaves. BF, bright field. Scale bars represent 50 μm. **(D)** Pull-down assays showing that SPL9 directly interacts with ABI5 *in vitro*. Purified MBP-SPL9 proteins could be pulled down by ABI5-GST proteins. MBP was used as a negative control. Arrowhead indicates specific bands. PD, pull down. **(E)** Schematic representation of the full length as well as truncated versions of SPL9 proteins. The N-terminal region of SPL9 contains the SBP domain. **(F)** LCI assays showing the interaction between the truncated SPL9 versions and full-length ABI5. **(G)** Schematic representation of the full length as well as truncated versions of ABI5 proteins. The conserved domains of ABI5 are depicted as I, II, and III (Bensmihen et al., 2002; Lopez-Molina et al., 2003); C-terminal region of ABI5 contains the bZIP domain. **(H)** LCI assays showing the interaction between the truncated ABI5 versions and full-length SPL9.

Since SPL9 can directly activate the transcription of ABA-responsive genes, we wondered whether SPL9 interacts with the master transcription factors of ABA signaling, such as ABI3, ABI4 and ABI5. To this end, we performed firefly luciferase complementation imaging (LCI) assays in *N. benthamiana* leaves. ABI3, ABI4, and ABI5 were fused with nLUC to produce nLUC-ABI3/ABI4/ABI5, respectively; meanwhile, SPL9 was fused with cLUC to generate cLUC-SPL9. LCI assays showed that strong luminescence signals were observed in the co-expressed samples of nLUC-ABI5 and cLUC-SPL9, but not in the samples of nLUC-ABI3/cLUC-SPL9, and nLUC-ABI4/cLUC-SPL9 (Figure 2A), indicating that SPL9 specifically interacts with ABI5. Furthermore, we conducted co–immunoprecipitation (Co-IP) assays using Col-0 and *GFP-rSPL9* seedlings with ABI5 antibody to confirm the interaction of SPL9 and ABI5. The results showed that ABI5 proteins were co-immunoprecipitated by SPL9 in *GFP-rSPL9* seedlings (Figure 2B), suggesting that SPL9 physically interacts with ABI5 *in vivo*. To further confirm the physical interaction between SPL9 and ABI5, we performed bimolecular fluorescence complementation (BiFC) assays in *N. benthamiana* leaves. SPL9 was fused with the C-terminal part of yellow fluorescent protein (cYFP), and ABI5 was fused with the N-terminal part of YFP (nYFP) to generate cYFP-SPL9 and nYFP-ABI5, respectively. The results illustrated that co-expression of cYFP-SPL9 and nYFP-ABI5 produced strong YFP fluorescence in the nucleus, whereas no YFP signal was observed in negative controls (Figure 2C). Finally, the pull down assays revealed that GST-ABI5 fusion proteins could retain MBP-SPL9, whereas GST alone could not (Figure 2D), suggesting that SPL9 could directly interact with ABI5 *in vitro*. As expected, different approaches demonstrated that SPL3 also interacts with ABI5 (Supplementary Figures 3A–C).

### Mapping of the Regions Required for the Interaction Between SPL9 and ABI5

To determine which regions of SPL9 are responsible for the interaction with ABI5, we performed LCI assays in *N. benthamiana*. SPL9 was divided into two truncated parts (N: amino-terminal, containing the intact SBP-box domain; C: carboxyl-terminal), according to the position of the highly conserved SBP domain (Figure 2E). The results showed that both the N and C termini of SPL9 interact with ABI5 (Figure 2F).

Next, to map which region of ABI5 is responsible for the interaction with SPL9, we generated different ABI5 derivatives, including ABI5-N (1-220 aa), ABI5-M (221-349 aa), and ABI5-C (350-442 aa) (Figure 2G), based on the highly conserved domains contained in ABI5 (Bensmihen et al., 2002; Lopez-Molina et al., 2003). The results showed that the middle region of ABI5 mediates its interaction with SPL9 (Figure 2H).

### ABI5 Is Required for the Function of SPL9 in Activating ABA Responses

**FIGURE 3:**
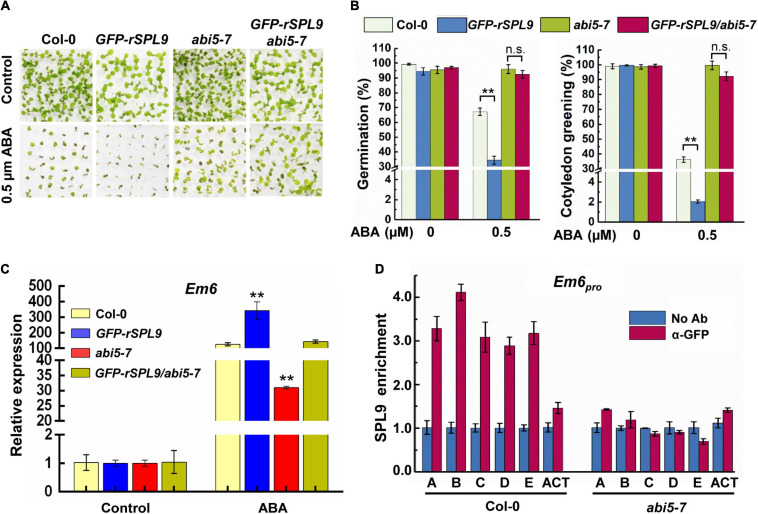
SPL9 promotes ABA responses in an ABI5-dependent manner. **(A)** Germination phenotypes of the indicated seedlings grown on medium without or with 0.5 μM ABA for 7 days. **(B)** Quantification of seed germination and cotyledon greening of indicated genotypes in response to ABA. Seed germination percentage was recorded at 3 days after the end of stratification. Cotyledon-greening percentage was recorded at 5 days after the end of stratification. Data shown are mean ± SD (*n* = 3). At least 100 seeds per genotype were measured in each replicate. **P < 0.01, n.s. indicates no significant difference (Student’s *t*-test). **(C)** qRT-PCR assays showing the expression levels of ABA-responsive gene in the indicated genotypes with ABA treatment. The 4-day-old seedlings were treated without or with 10 μM ABA for 4 h. The expression levels of *Em6* in untreated seedlings (Control) for each genotype were set to one. Data are means ± SD (*n* = 3). **P < 0.01 (Student’s *t*-test). **(D)** ChIP-qPCR assays showing that the ABA-triggered enrichment of SPL9 on the *Em6* promoter is dependent on ABI5. The 6-day-old *GFP-rSPL9* and *GFP-rSPL9/abi5-7* seedlings treated with 50 μM ABA for 2 h were harvested for ChIP assays. Error bars denote ± SD (*n* = 3). *ACT7* was used as a control.

To evaluate the functional relationship between SPL9 and ABI5 in regulating ABA responses, we generated the *GFP-rSPL9/abi5-7* plants via genetic crossing and examined their seed germination and cotyledon greening phenotypes in response to ABA treatment. Consistent with the above results (Figure 1A and Supplementary Figure 1), the *GFP-rSPL9* seedlings displayed an ABA-hypersensitive phenotype, whereas the *GFP-rSPL9/abi5-7* and *abi5-7* seedlings displayed decreased sensitivities to ABA treatment compared with Col-0 in terms of seed germination and cotyledon greening (Figures 3A,B). This genetic evidence supports the notion that SPL9 enhances ABA responses in an ABI5-dependent manner.

We subsequently determined whether the SPL9-mediated up-regulation of ABA-responsive gene expression is also dependent on ABI5. As expected, the ABA-induced expression of *Em1* and *Em6* in the *GFP-rSPL9* seedlings was significantly increased compared with Col-0, whereas their expression was markedly decreased in the *abi5-7* mutant (Figure 3C and Supplementary Figure 4A). Intriguingly, the SPL9-enhanced expression of *Em1* and *Em6* was completely suppressed by the *abi5-7* mutation (Figure 3C and Supplementary Figure 4A). These results promote us to conclude that SPL9 activates the expression of ABA-responsive genes in an ABI5-dependent manner.

### The Enrichment of SPL9 at the ABA-Responsive Genes Is Dependent on ABI5

Since ABI5 is required for the function of SPL9 in activating ABA responses, we wondered whether the enrichment of SPL9 at the promoters of ABA-responsive genes is also dependent on ABI5. To this end, we performed ChIP-qPCR assays using the 6-day-old *GFP-rSPL9* and *GFP-rSPL9/abi5-7* seedlings treated with 50 μM ABA for 2 h. Interestingly, the results showed that the ABA-triggered enrichment of SPL9 at the promoters of *Em1* and *Em6* was reduced in the *abi5-7* mutant compared with that in the wild type (Figure 3D and Supplementary Figure 4B). Notably, the GFP-SPL9 protein levels did not show detectable difference between the wild type and *abi5-7* mutant with or without ABA treatment (Supplementary Figure 5). Therefore, we propose that ABI5 facilitates the ABA-triggered recruitment of SPL9 into the chromatin regions of ABA-responsive genes.

### SnRK2s Interact With SPLs

**FIGURE 4:**
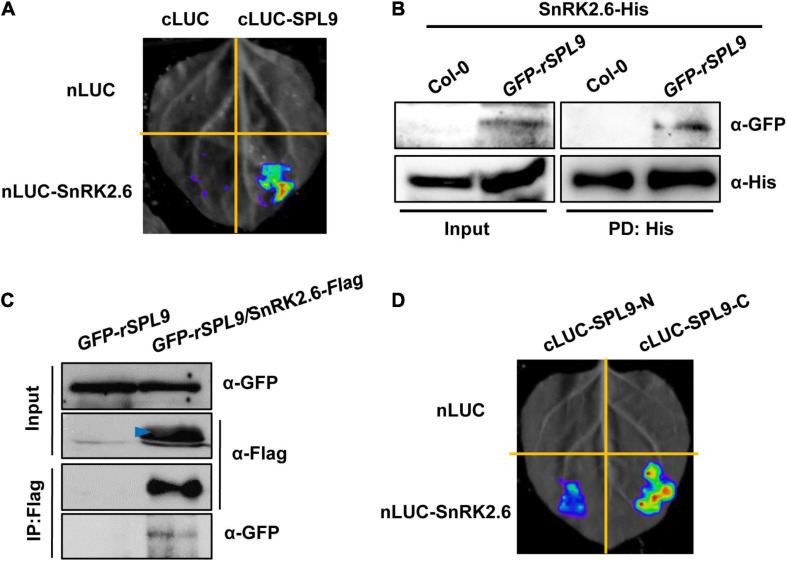
SnRK2.6 directly interacts with SPL9. **(A)** LCI assays showing the interaction between SnRK2.6 and SPL9 in *N. benthamiana* leaves. Empty vectors were used as negative controls. **(B)** Semi*-in vivo* pull-down assays showing the interaction of SnRK2.6 and SPL9. Anti-GFP and anti-His antibodies were used for immunoblotting assays. PD, pull down. **(C)** Co-IP assays showing that SnRK2.6 physically interacts with SPL9 *in vivo*. Total proteins were analyzed by immunoblotting with anti-Flag and anti-GFP antibodies. Arrowhead indicates specific band. **(D)** LCI assays showing the interaction between the truncated SPL9 versions and full-length SnRK2.6.

Considering the facts that SnRK2s can interact with and phosphorylate ABI5, and SPLs also interact with ABI5, we were curious whether SnRK2s interact with and phosphorylate SPLs. To this end, LCI assays were performed in *N. benthamiana* leaves. As shown in Figure 4A and Supplementary Figure 6, strong LUC activity was exclusively observed in the co-expressed samples of nLUC-SnRK2s and cLUC-SPL9, indicating that SnRK2s could physically interact with SPL9. We next conducted the semi-*in vitro* pull down assays using the *GFP-rSPL9* seedlings and SnRK2.6-His proteins. The results showed that the GFP-SPL9 fusion proteins were pulled down by SnRK2.6-His proteins (Figure 4B). Furthermore, we generated the *GFP-rSPL9/SnRK2.6-Flag* double transgenic plants for Co-IP assays. As shown in Figure 4C, the GFP-SPL9 fusion proteins were immunoprecipitated by SnRK2.6-Flag, suggesting that SnRK2.6 interacts with SPL9 *in vivo*. Taken together, these results demonstrate that SnRK2.6 directly interacts with SPL9 *in vitro* and *in vivo*. Meanwhile, SnRK2s could also interact with SPL3 (Supplementary Figure 7).

Next, to map which region of SPL9 is responsible for its interaction with SnRK2.6, the full-length SPL9 protein was divided into two parts as described above (Figure 2E). The LCI assays in *N. benthamiana* leaves showed that the C terminus of SPL9 predominately mediates the interaction with SnRK2.6 (Figure 4D).

### SnRK2.6 Phosphorylates SPLs

**FIGURE 5:**
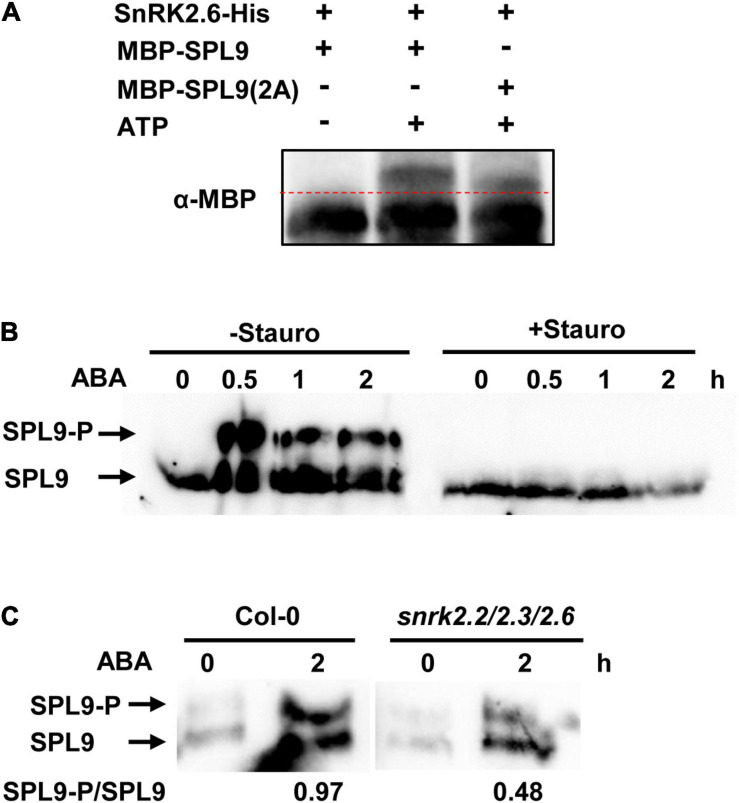
SnRK2.6 phosphorylates SPL9 *in vitro* and *in vivo*. **(A)** *In vitro* kinase assays for the SPL9 protein by SnRK2.6 using phos-tag gel. The mutated SPL9^*S203A,S281A*^ protein is abbreviated as SPL9(2A). Proteins were detected by immunoblotting with anti-MBP antibody. **(B)** *In vivo* phosphorylation assays for SPL9 using phos-tag gel. The 6-day-old *GFP-rSPL9* seedlings were treated with 50 μM ABA and without or with 5 μM Staurosporine for indicated times. Proteins were analyzed by immunoblotting with anti-GFP antibody. Stauro, Staurosporine. **(C)** *In vivo* phosphorylation assays for SPL9 in indicated genotypes using phos-tag gel. The 6-day-old *GFP-rSPL9* and *GFP-rSPL9*/*snrk2.2*/*2.3*/*2.6* seedlings were treated without or with 50 μM ABA for 2 h. Proteins were analyzed by immunoblotting with anti-GFP antibody. The arrows in **(B)** and **(C)** indicate the phosphorylated or unphosphorylated SPL9.

Since the protein kinase SnRK2.6 interacts with SPL9, we would like to test whether SPL9 is a substrate of SnRK2.6. It has been reported that SnRK2s usually phosphorylate the Ser/Thr residues in the RXXS/T motifs of their substrates (Kobayashi et al., 2005). In this scenario, we first searched the RXXS/T motifs in the SPL9 protein sequence. We found that SPL9 contains two conserved RXXS motifs with putative phosphorylation sites Ser203 and Ser281 (Supplementary Figure 8). The *in vitro* phosphorylation assays with the Phos-tag approach showed that SPL9 could be evidently phosphorylated by SnRK2.6 (Figure 5A). Further, we substituted the two putative SnRK2.6 phosphorylation sites of SPL9 with Ala (non-phosphorylated form) to generate the SPL9^*S*203*A,S*281*A*^ mutant form [SPL9(2A)] for *in vitro* phosphorylation assays. As shown in Figure 5A, the phosphorylation band of SPL9(2A) was weaker and migrated faster compared with that of the SPL9 protein, indicating that the Ser203 and Ser281 residues are two major SnRK2.6 phosphorylation sites of SPL9. Meanwhile, SnRK2.6 could also phosphorylate SPL3 *in vitro* (Supplementary Figure 9).

Furthermore, we wondered whether ABA regulates the SnRK2s-mediated phosphorylation of SPL9 protein. To this end, we used the 6-day-old *GFP-rSPL9* seedlings treated with 50 μM ABA for different time points. Phos-tag gel assays showed that the phosphorylated SPL9 proteins obviously accumulated from 0.5 h after ABA treatment, suggesting that ABA treatment promotes the phosphorylation of SPL9 *in vivo* (Figure 5B). Significantly, the ABA-triggered accumulation of phosphorylated SPL9 proteins was almost abolished by the treatment of staurosporine, a general Ser/Thr-kinase inhibitor (Figure 5B). To further verify whether the ABA-induced phosphorylation of SPL9 is dependent on the SnRK2s protein kinases, we generated the *GFP-rSPL9/snrk2.2/2.3/2.6* plants by genetic crossing. Phos-tag gel assays showed that the ABA-induced phosphorylation band of SPL9 proteins in the *snrk2.2/2.3/2.6* triple mutant background was much weaker than that in the Col-0 background (Figure 5C). These observations suggest that SnRK2s are required for the ABA-induced phosphorylation of SPL9.

### SnRK2-Mediated Phosphorylation Is Required for the Function of SPL9 in Enhancing ABA Responses

**FIGURE 6:**
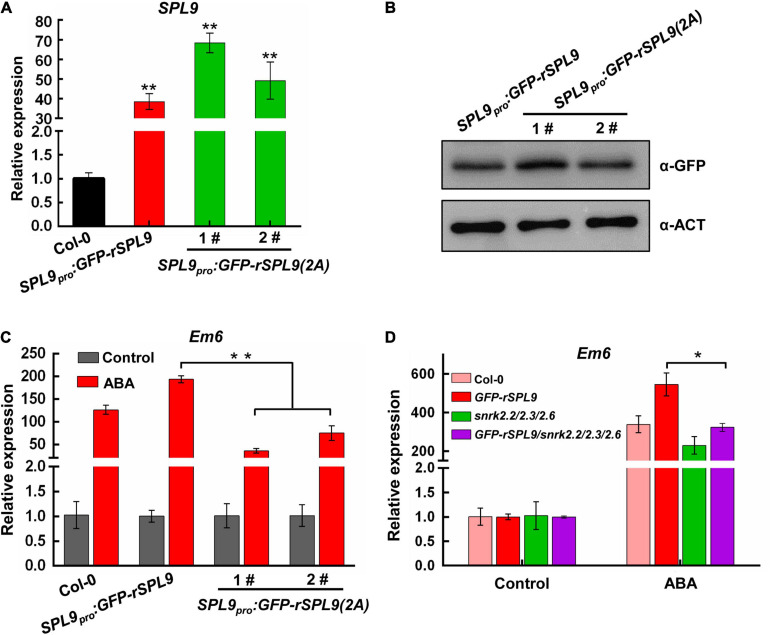
The phosphorylation by SnRK2s is required for SPL9 in enhancing ABA responses. **(A)** qRT-PCR assays showing the expression levels of SPL9 in the indicated seedlings. Values are means ± SD (*n* = 3). **P < 0.01 by the Student’s *t*-test. **(B)** Immunoblotting assays showing the protein levels of SPL9 in the indicated seedlings. Anti-GFP antibody was used. Actin was used as a loading control. **(C)** qRT-PCR assays showing that phosphorylation of SPL9 by SnRK2s is required for the activation of *Em6* expression. The 6-day-old seedlings were treated without or with 50 μM ABA for 4 h. The expression levels of *Em6* in control samples were set to 1 for each genotype. Data are means ± SD (*n* = 3). **P < 0.01 (Student’s *t*-test). **(D)** qRT-PCR assays showing that the SPL9-mediated ABA induction of *Em6* is dependent on SnRK2s. The 4-day-old seedlings were treated without or with 10 μM ABA for 4 h. The expression levels of *Em6* in control samples were set to one for each genotype. Data are means ± SD (*n* = 3). **P < 0.01 (Student’s *t*-test).

To elucidate the biological significance of SPL9 phosphorylation by SnRK2s in regulating ABA responses, we generated the *SPL9_pro_:GFP-rSPL9* and *SPL9_pro_:GFP-rSPL9(2A)* transgenic plants. We chose the *SPL9_pro_:GFP-rSPL9* and *SPL9_pro_:GFP-rSPL9(2A)* transgenic lines with similar SPL9 expression levels for further phenotypic analyses (Figures 6A,B). As expected, the induction of ABA-responsive genes by ABA in the *SPL9_pro_:GFP-rSPL9* seedlings was higher than that in the wild type (Figure 6C and Supplementary Figure 10A). Significantly, we found that the ABA induction of ABA-responsive genes in the *SPL9_pro_:GFP-rSPL9(2A)* seedlings was lower than that in the *SPL9_pro_:GFP-rSPL9* seedlings (Figure 6C and Supplementary Figure 10A), suggesting that the phosphorylation is critical for the function of SPL9 in enhancing ABA responses. To further determine whether SnRK2s is required for the function of SPL9 in enhancing ABA responses, we examined the ABA-induced expression levels of *Em1* and *Em6* in the Col-0, *GFP-rSPL9*, *snrk2.2/2.3/2.6* and *GFP-rSPL9/snrk2.2/2.3/2.6* seedlings. Our results showed that the SPL9-enhanced expression of ABA-responsive genes was abolished in the *snrk2.2/2.3/2.6* triple mutants compared to the wild type (Figure 6D and Supplementary Figure 10B). The above observations demonstrate that SnRK2s-mediated phosphorylation is required for the activity of SPL9 in enhancing ABA responses. In addition, our results showed the SnRK2s-mediated phosphorylation did not affect the subcellular localization of SPL9 protein in plant cells (Supplementary Figure 11).

## Discussion

Accumulating evidences have shown that the miR156-SPL regulatory module is highly conserved among different land plant species, and plays important roles in regulating diverse plant developmental processes (Wang and Wang, 2015). Nevertheless, its roles in the ABA signaling remain largely unknown. In this study, we uncover a new biological role of the miR156-SPLs module in regulating ABA response and elucidate the underlying mechanism.

### SPLs Activate ABA Signaling in an ABI5-Dependent Manner

The miR156-targeted SPLs have been shown to regulate plant hormone signaling through interacting with several transcription regulators. For example, SPL9 interacts with ARR2, a transcriptional activator of cytokinin signaling, to repress cytokinin response and shoot regeneration (Zhang et al., 2015); SPL9 also interacts with RGA, a transcription repressor of gibberellin (GA) signaling, to regulate flowering time (Yu et al., 2012). However, the role of SPL9 in ABA signaling remains unknown.

In this study, we provide several lines of evidence to demonstrate that the miR156-targeted SPLs facilitate ABA signaling through the interaction with ABI5, a master transcription factor in ABA signaling. First, the miR156-targeted SPLs positively regulate ABA responses (Figures 1A,C and Supplementary Figure 1). Second, ABA treatment facilitates the recruitment of SPL9 to the promoters of ABA-responsive genes (Figure 1D and Supplementary Figure 2A). Third, SPLs physically interacts with ABI5 (Figure 2). Fourth, genetic analyses reveal that ABI5 is functionally required for SPL9 in activating ABA responses (Figures 3A–C and Supplementary Figure 4A). Fifth, the ABA-induced enrichment of SPL9 at the promoters of ABA-responsive genes is largely dependent on ABI5 (Figure 3D and Supplementary Figure 4B). The above-described action mode of SPL9 suppose that SPL9 might function as a cofactor of ABI5 to promote ABA responses. Thus, it is conceivable that the SPLs-mediated enhancement of ABA responses might offer an advantageous strategy for plants to adapt stressful conditions.

### SnRK2s Phosphorylate and Activate SPLs During ABA Responses

The SnRK2s family protein kinases act through activation of the transcriptional activity of ABI5 by phosphorylation to promote ABA responses (Nakashima et al., 2009). In this study, we showed that SnRK2s physically interact with and phosphorylate SPLs (Figures 4, 5 and Supplementary Figures 6–9). We further focused on the biological relevance of the phosphorylation of SPL9 by SnRK2s in regulating ABA responses. We found that the expression levels of ABA-responsive genes in the *SPL9_pro_:GFP-rSPL9(2A)* seedlings was lower than that in the *SPL9_pro_:GFP-rSPL9* seedlings under ABA treatment (Figure 6C and Supplementary Figure 10A). Notably, both the protein levels and subcellular localization of SPL9 seem not to be affected by the SnRK2s-medidated phosphorylation (Figure 6B and Supplementary Figure 11). Moreover, genetic analyses showed that the SPL9-mediated ABA induction of ABA-responsive genes was abolished in the absence of *SnRK2.2/2.3/2.6* (Figure 6D and Supplementary Figure 10B). Taken together, we conclude that the phosphorylation by SnRK2s is essential for SPLs in promoting ABA responses.

### The SnRK2s-SPLs-ABI5 Module Is Critical for ABA Signaling

**FIGURE 7:**
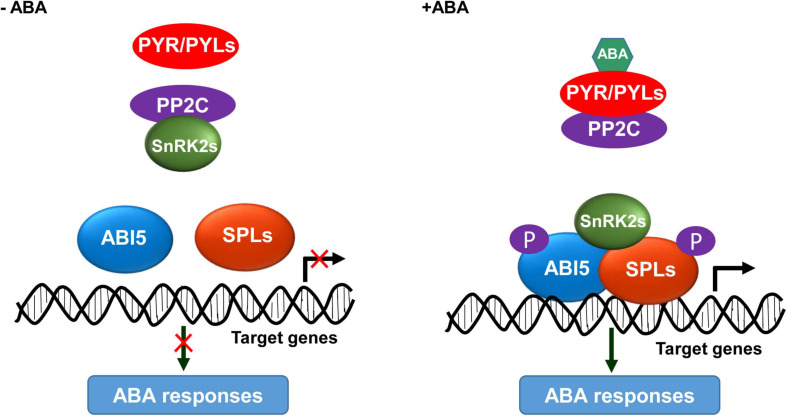
A proposed working model for the SnRK2s-SPLs-ABI5 module in activating ABA responses. In the absence of ABA, PP2C interacts with and dephosphorylates SnRK2s; consequently, ABI5 and SPLs are inactive and unable to activate the ABA responses. In the presence of ABA, its receptors PYR/PYLs interact with PP2C to release the inhibition on SnRK2s activity; thereby, SnRK2s interact with and phosphorylate ABI5 and SPLs, leading to their enrichment at the promoter of target genes to activate ABA responses.

Based on our findings and previous studies (Fujii et al., 2009; Chen et al., 2020), we propose a working model for the mechanism of SnRK2s-SPLs-ABI5 module in activating ABA responses. In the absence of ABA, PP2C dephosphorylates and inactivates SnRK2s; consequently, SPLs and ABI5 are inactive and unable to activate the downstream genes required for ABA responses (Figure 7). In the presence of ABA, its receptors PYR/PYLs interact with PP2C to release the inhibition on SnRK2s activity; thereby, the ABA-activated SnRK2s interact with and phosphorylate SPLs and ABI5, leading to their enrichments on the promoter of target genes to activate ABA responses (Figure 7). In summary, we discovered that the SnRK2s-SPLs-ABI5 regulatory module represents a signaling hub mediating the enhancement of ABA signaling for plants to adapt to stressful conditions.

### Phosphorylation of SPLs in Response to Different Stimuli

Previous studies reported that the *Ideal Plant Architecture 1/Wealthy Farmer’s Panicle* (*IPA1/WFP*) gene, encoding an OsSPL14 transcription factor in rice, plays an important role in regulating plant architecture (Jiao et al., 2010; Miura et al., 2010). In addition, the fungus *Magnaporthe oryzae* infection can induce the phosphorylation of OsSPL14, consequently alter its DNA binding specificity (Wang et al., 2018). Unfortunately, the specific protein kinase responsible for the phosphorylation of OsSPL14 in response to *M. oryzae* infection remains to be identified. Significantly, we here found that ABA treatment can induce the phosphorylation of SPL9 by SnRK2s to amplify ABA responses in *Arabidopsis*. Notably, the ABA-induced phosphorylation of SPL9 was reduced in the *snrk2.2*/*2.3*/*2.6* triple mutants rather than completely abolished as shown in the wild type seedlings treated with a general Ser/Thr-kinase inhibitor staurosporine, indicating that there might be other protein kinases could phosphorylate SPLs *in vivo*. Thus, we propose that the plant-specific transcription factors SPLs may be phosphorylated and functionally modulated by different protein kinases in response to endogenous cues and external challenges.

## Data Availability Statement

The original contributions presented in the study are included in the article/Supplementary Material, further inquiries can be directed to the corresponding author/s.

## Author Contributions

JS designed the research. HD, SY, and YJ performed the experiments. JS, HD, SY, RY, YZhang, YZhou, and YZhu analyzed the data. JS, HD, and SY wrote the manuscript. JS and YZhu revised the manuscript. All authors contributed to the article and approved the submitted version.

## Conflict of Interest

The authors declare that the research was conducted in the absence of any commercial or financial relationships that could be construed as a potential conflict of interest.

## Supplementary Material

Supplementary Figure 1 The miR156-targeted SPLs positively regulate ABA responses during seed germination and cotyledon greening.

Supplementary Figure 2 SPL9 directly activates the transcriptional expression of *Em1*.

Supplementary Figure 3 SPL3 directly interacts with ABI5.

Supplementary Figure 4 SPL9 activates the expression of *Em1* in an ABI5-dependent manner.

Supplementary Figure 5 The GFP-SPL9 protein levels were comparable between the *GFP-rSPL9* and *GFP-rSPL9/abi5-7* seedlings.

Supplementary Figure 6 SnRK2s physically interact with SPL9.

Supplementary Figure 7 SnRK2s physically interact with SPL3.

Supplementary Figure 8 The putative SnRK2.6 phosphorylation sites in SPL9 protein.

Supplementary Figure 9 *In vitro* kinase assay showing that SnRK2.6 could phosphorylate SPL3.

Supplementary Figure 10 Phosphorylation by SnRK2s is required for SPL9-activated expression of *Em1*.

Supplementary Figure 11 SnRK2s-mediated phosphorylation did not affect the nuclear localization of SPL9.

Supplementary Table 1 Primers used for DNA constructs in this study.

Supplementary Table 2 Constructs used in this study.

Supplementary Table 3 Primers used for qRT-PCR.

Supplementary Table 4 Primers used for ChIP-qPCR assays.
