# Supplementary material for: MIR156-Targeted SPL9 Is Phosphorylated by SnRK2s and Interacts With ABI5 to Enhance ABA Responses in Arabidopsis
Source: Front Plant Sci. 2021 Jul 21;12:708573. doi: 10.3389/fpls.2021.708573 (PMC8334859; doi:10.3389/fpls.2021.708573)
Supplement: Supplementary Figure 1 — The miR156-targeted SPLs positively regulate ABA responses during seed germination and cotyledon greening. [file Data_Sheet_1.PDF]

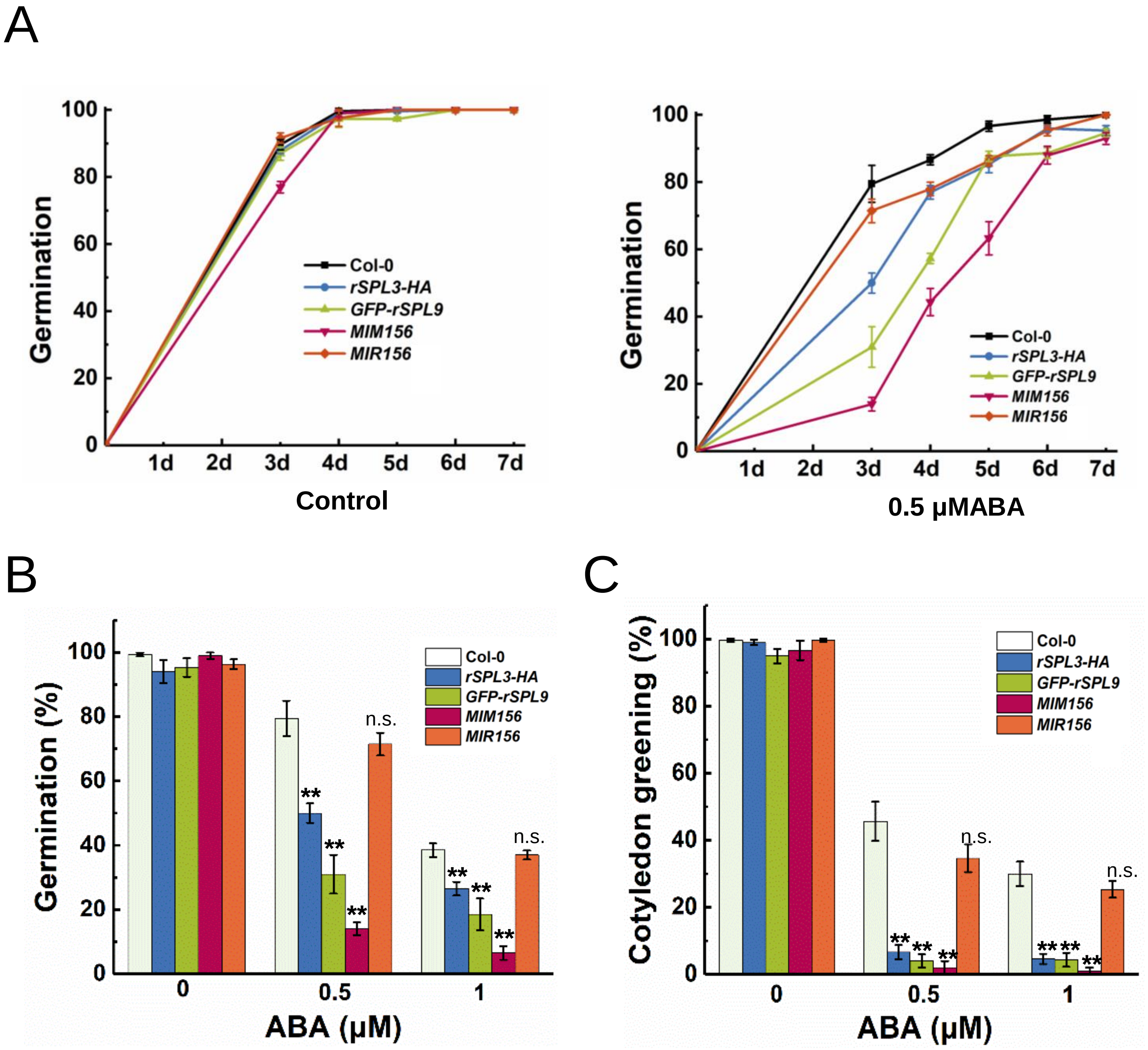

Supplementary Figure 1 | The miR156-targeted SPLs positively regulate ABA responses during seed germination and cotyledon greening. A Seed germination percentages of indicated genotypes grown on medium without or with 0.5 μM ABA during a time course. **B, C** Quantification of seed germination **(B)** and cotyledon greening **(C)** of indicated genotypes in response to different concentration of ABA. Seed germination percentage was recorded at 3 d after the end of stratification and cotyledon-greening percentage was recorded at 5 d after the end. Data shown are mean ± SD (n = 3). At least 100 seeds per genotype were measured in each replicate. ***P* < 0.01, n.s. indicates no significant difference (Student's *t*-test).

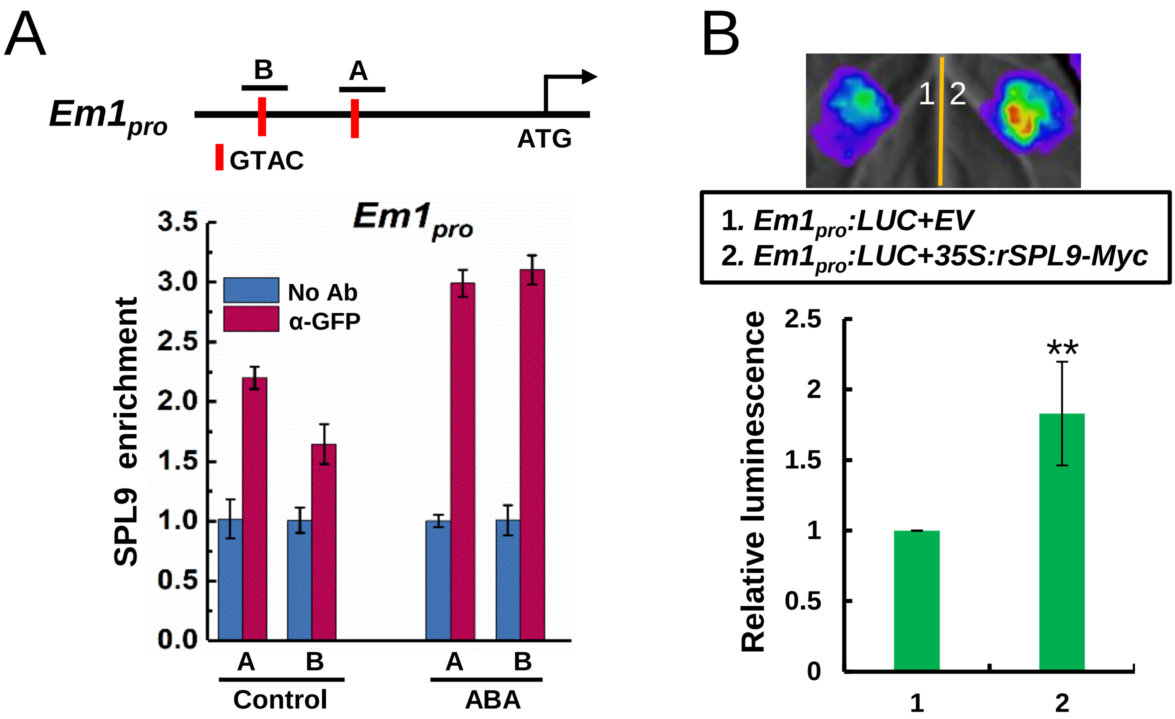

Supplementary Figure 2 | SPL9 directly activates the transcriptional expression of *Em1*. A ChIP-qPCR analysis showing enrichment of SPL9 on the *Em1* promoter region. Red boxes in the upper panel indicate the positions of SBP-box binding core motifs. The 6-d-old *GFP-rSPL9* seedlings treated without (Control) or with 50 μM ABA for 2 hours were harvested for ChIP assays. Error bars denote ± SD (n = 3). **B** Transient expression assays showing the activation of *Em1* promoter by SPL9. Upper panel shows a representative leaf image, and the column diagram represents the quantification of the relative luminescence intensities (n = 10). The mean value in combination 1 was set to 1. $^{**}P < 0.01$ (Student's *t*-test).

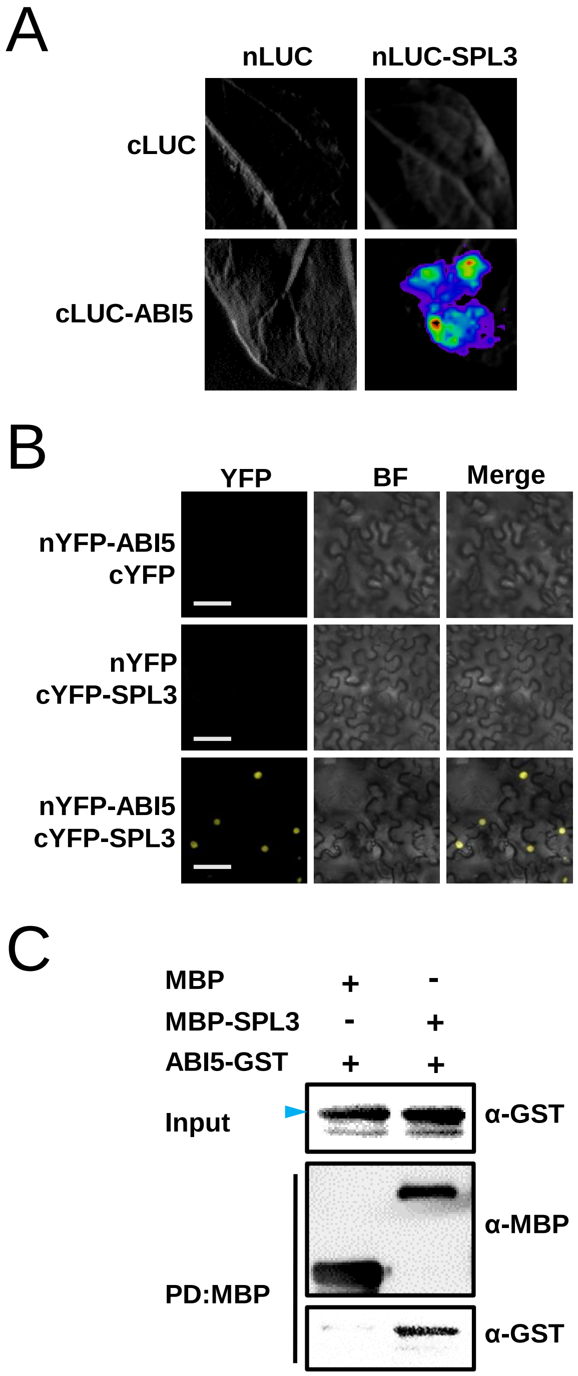

Supplementary Figure 3 | SPL3 directly interacts with ABI5. A LCI assays showing that SPL3 interacts with ABI5 in *N. benthamiana* leaves. **B** BiFC assays showing the interaction of SPL3 and ABI5 in *N. benthamiana* leaves. **C** Pull-down assays showing that SPL3 directly interacts with ABI5 *in vitro* . The ABI5-GST fusion proteins could be pulled down by MBP-SPL3 proteins but not MBP alone. Arrowhead indicates specific bands. PD, pull down.

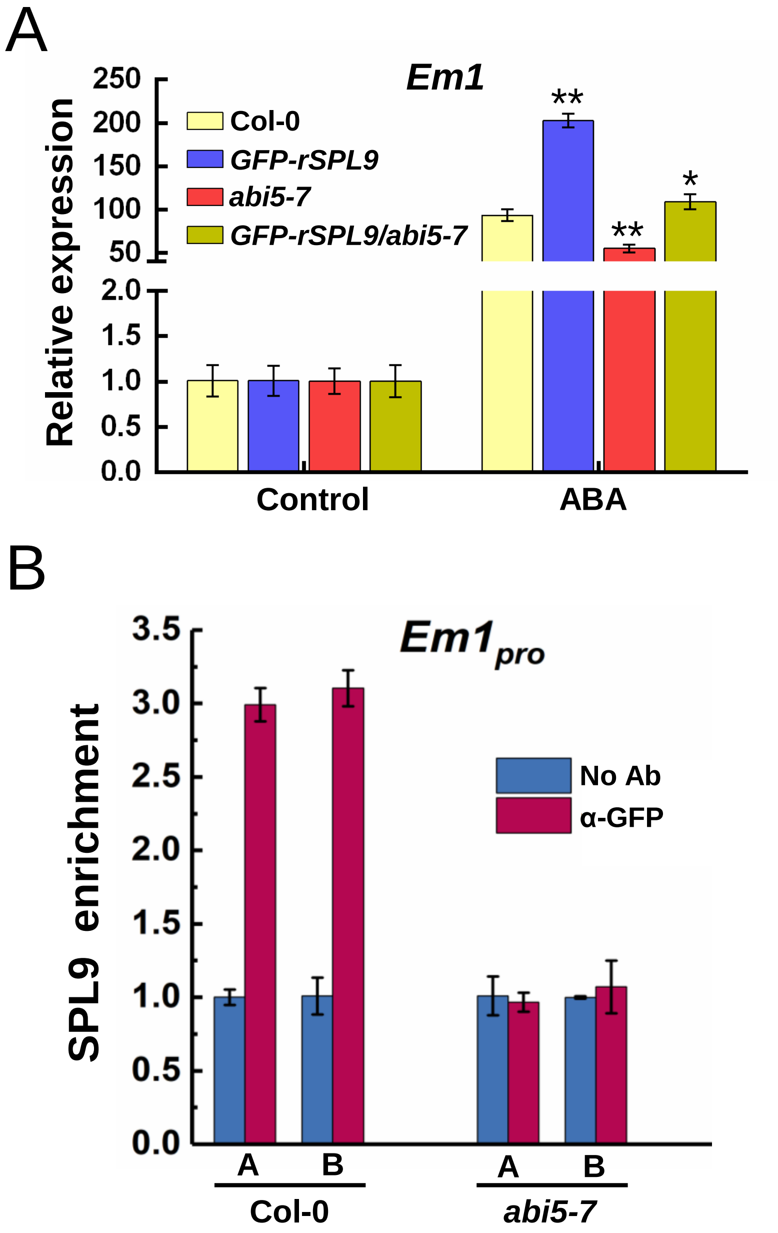

Supplementary Figure 4 | SPL9 activates the expression of *Em1* in an ABI5-dependent manner. **A** qRT-PCR assays showing the expression levels of *Em1* in the indicated genotypes in response to ABA treatment. The 4-d-old seedlings were treated without or with 10 μM ABA for 4 h. The expression levels of *Em1* in untreated seedlings (Control) for each genotype were set to 1. Data are means ± SD (n = 3). **P* < 0.05, ***P* < 0.01 (Student's *t*-test). **B** ChIP-qPCR analysis showing that the ABA-induced enrichment of SPL9 on the *Em1* promoter regions is dependent on ABI5. The 6-d-old *GFP-rSPL9* and *GFP-rSPL9/abi5-7* seedlings were treated with 50 μM ABA for 2 h and then harvested for ChIP assays. Error bars denote ± SD (n = 3).

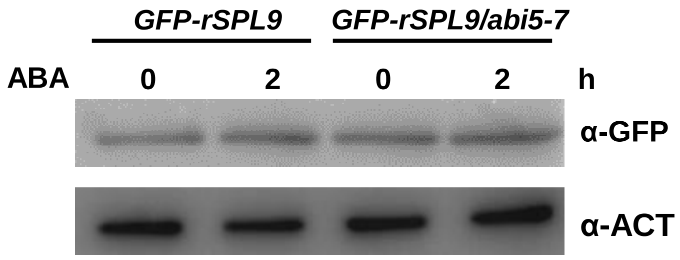

Supplementary Figure 5 | The GFP-SPL9 protein levels were comparable between the *GFP-rSPL9* and *GFP-rSPL9/abi5-7* seedlings. Immunoblotting assays showing the protein levels of SPL9 in the indicated 6-d-old seedlings treated without or with 50 μM ABA for 2 h. Actin was used as a loading control.

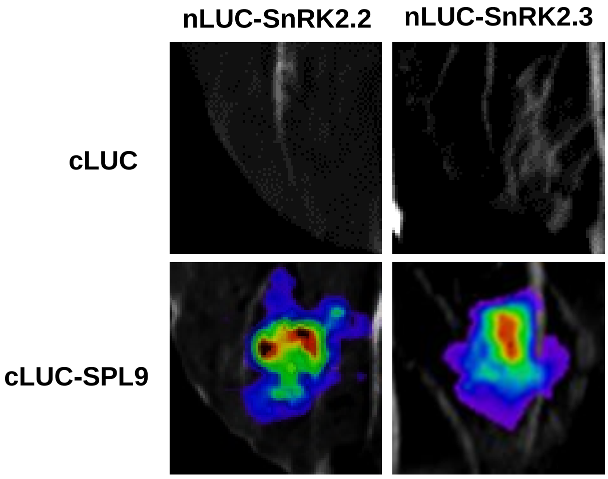

Supplementary Figure 6 | SnRK2s physically interact with SPL9. LCI assays showing that SnRK2s physically interact with SPL9. The nLUC-SnRK2s and cLUC-SPL9 constructs were co-transformed into leaves of *N. benthamiana*. Empty vectors were used as negative controls.

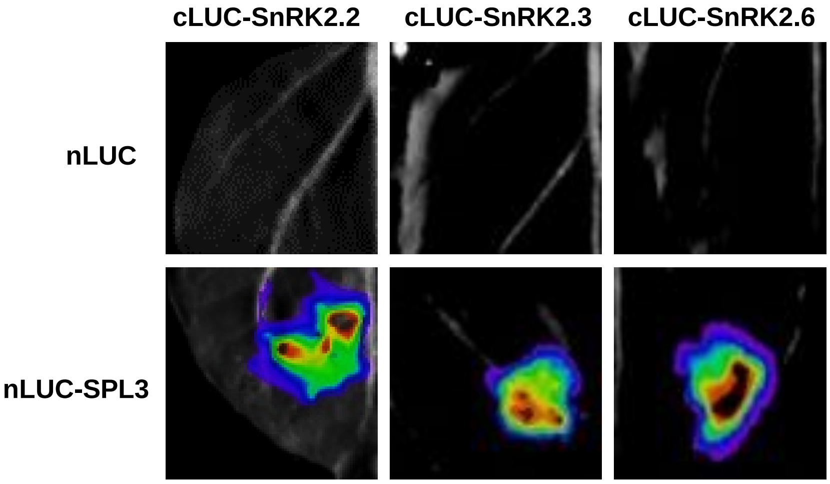

Supplementary Figure 7 | SnRK2s physically interact with SPL3. LCI assays showing that SnRK2s physically interact with SPL3. The cLUC-SnRK2s and nLUC-SPL3 constructs were co-transformed into leaves of *N. benthamiana*. Empty vectors were used as negative controls.

```
SPL9 :  MEMGSNSGPGHGPGQAESGGSSTESSSFSGGLMFGQKIYFEDGGGGSGSSSSGGRSNRRVRGG  :   63
SPL9 :  GSGQSGQIPRCQVEGCGMDLTNAKGYYSRHRVCGVHSKTPKVTVAGIEQRFCQQCSRFHQLPE  :  126
SPL9 :  FDLEKRSCRRRLAGHNERRRKPQPASLSVLASRYGRIAPSLYENGDAGMNGSFLGNQEIGWPS  :  189
SPL9 :  SRTLDTRVMRRPVSSPSWQINPMNVFSQGSVGGGTSFSSPEIMDTKLESYKGIGDSNCALSL  :  252
SPL9 :  LSNPHQPHDNNNNNNNNNNNNNNTWRASSGFGPMTVTMAQPPPAPSQHQYLNPPWVFKDNDND  :  315
SPL9 :  MSPVLNLGRYTEPDNCQISSGTAMGEFELSDHHHQSRRQYMEDENTRAYDSSSHHTNWSL-  :  375
```

Supplementary Figure 8 | The putative SnRK2.6 phosphorylation sites in SPL9 protein. Blue lines indicate the RXXS/T motifs of SPL9 protein, which contains two putative SnRK2.6 phosphorylation sites Ser203 and Ser281, as shown by red arrows.

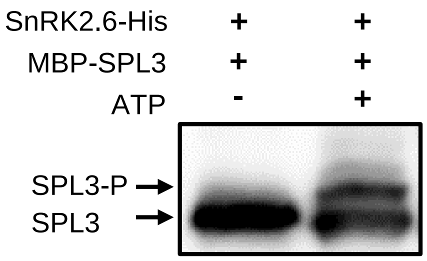

Supplementary Figure 9 | *In vitro* kinase assay showing that SnRK2.6 could phosphorylate SPL3.

Proteins were detected by phos-tag gel with anti-MBP antibody.

A

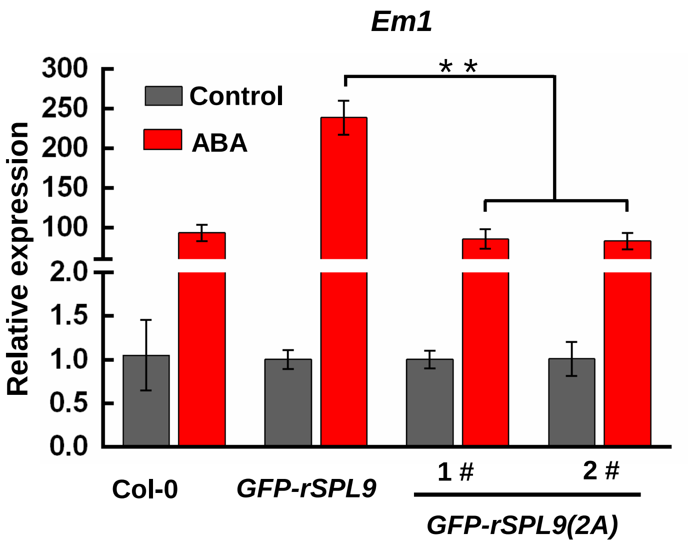

B

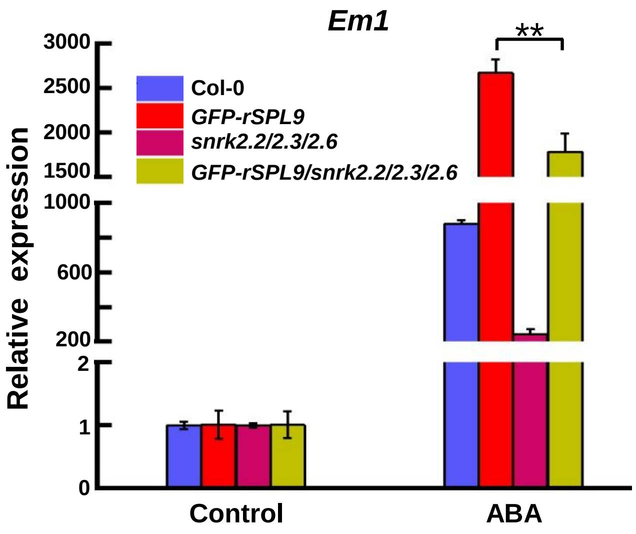

Supplementary Figure 10 | Phosphorylation by SnRK2s is required for SPL9-activated expression of *Em1*. A qRT-PCR assays showing phosphorylation by SnRK2s is required for SPL9-mediated activation of *Em1*. The 6-d-old seedlings were treated without or with 50 μM ABA for 4 h. The expression of *Em1* in control samples was set to 1 for each genotype. Data are means ± SD (n = 3). $^{**}P < 0.01$ (Student's *t*-test). **B** qRT-PCR assays showing the activation of *Em1* by SPL9 is dependent on SnRK2s. The 4-d-old seedlings were treated without or with 50 μM ABA for 4 h. The expression of *Em1* in control samples was set to 1 for each genotype. Data are means ± SD (n = 3). $^{**}P < 0.01$ (Student's *t*-test).

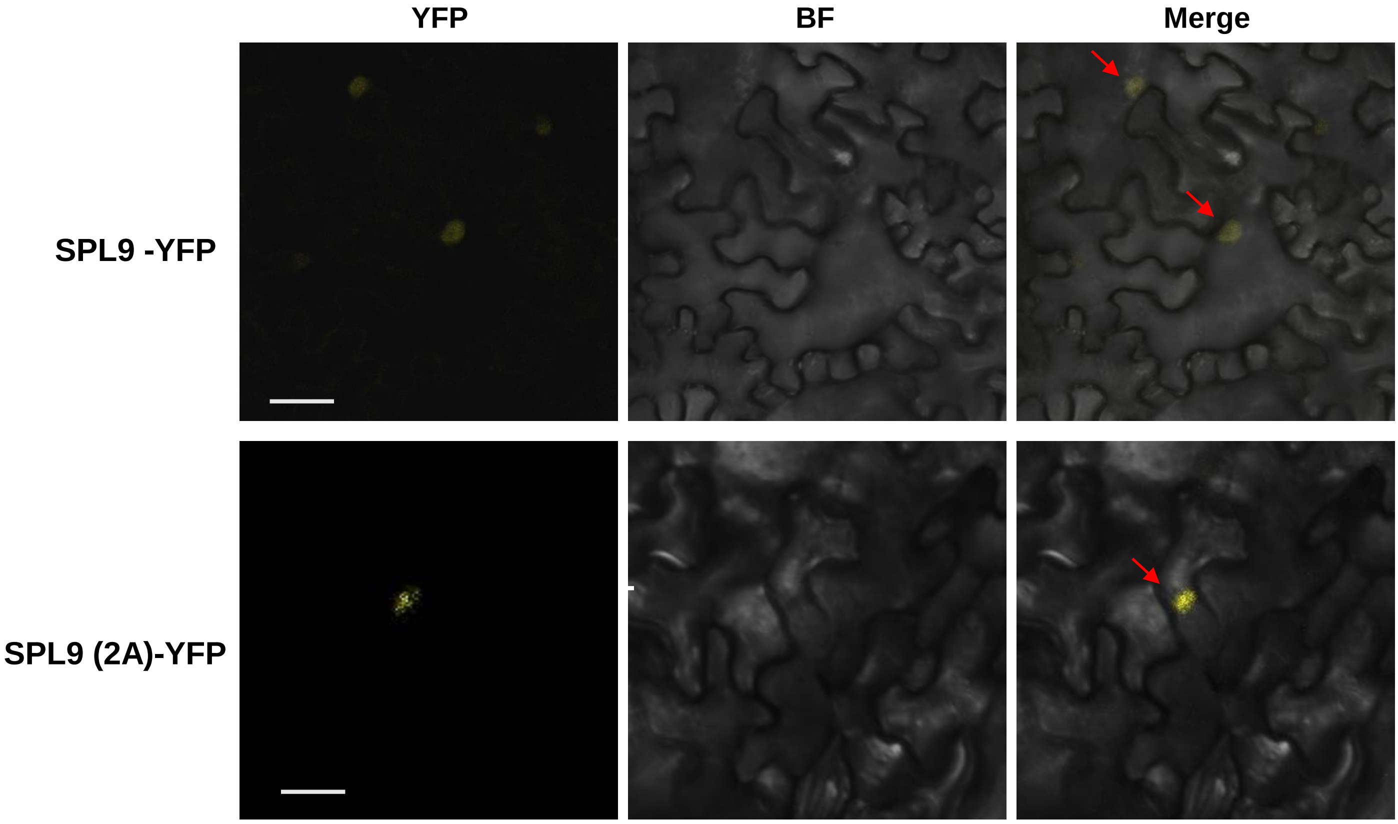

Supplementary Figure 11 | SnRK2s-mediated phosphorylation did not affect the nuclear localization of SPL9. Subcellular localization assays showed that both the SPL9 and SPL9(2A) mutant proteins were localized in nuclei.

Supplementary Table 1 | Primers used for DNA constructs in this study

Primer name	Forward primer (5'-3')	Reverse primer (5'-3')
nLUC-SPL9	GACGAGCTCGGTACCATGGAGATGGGTTCCAACT	CGAGATCTGGTCGACGAGAGACCAGTTGGTATGGTGA
cLUC-SPL9	TCCCGGGGCGGTACCATGGAGATGGGTTCCAACT	GTAGTCCATTTGTTGGAGAGACCAGTTGGTATGG
nLUC-SPL3	GGACGAGCTCGGTACCATGAGTATGAGAAGAAGCAA	ACGAGATCTGGTCGACGTCAGTTGTGCTTTTCCGCC
cLUC-ABI5	GTCCCGGGGCGGTACCATGGTAACTAGAGAAACGAA	TGTAGTCCATTTGTTGGAGTGGACAACTCGGGTTCC
nLUC-ABI5	GGGACGAGCTCGGTACCATGGTAACTAGAGAAACGAAGT	TACGAGATCTGGTCGACGAGTGGACAACTCGGGTTCCTC
nLUC-ABI3	GGGACGAGCTCGGTACCATGAAAAGCTTGCATGTGGCGG	TACGAGATCTGGTCGACTTTAACAGTTTGAGAAGTTGGT
nLUC-ABI4	GGGACGAGCTCGGTACCATGGACCCTTTAGCTTCCCAAC	TACGAGATCTGGTCGACATAGAATTCCCCCAAGATGGGA
nLUC-SnRK2.2	GACGAGCTCGGTACCATGGATCCGGCGACTAATTCAC	CGAGATCTGGTCGACGAGAGCATAAACTATCTCTCC
nLUC-SnRK2.3	GACGAGCTCGGTACCATGGATCGAGCTCCGGTGACC	CGAGATCTGGTCGACGAGAGCGTAAACTATCTCTCC
nLUC-SnRK2.6	GGGACGAGCTCGGTACCATGGATCGACCAGCAGTGAGTG	TACGAGATCTGGTCGACCATTGCGTACACAATCTCTCCG
cLUC-SnRK2.2	TCCCGGGGCGGTACCATGGATCCGGCGACTAATTCA	GTAGTCCATTTGTTGGAGAGCATAAACTATCTCTCC
cLUC-SnRK2.3	TCCCGGGGCGGTACCATGGATCGAGCTCCGGTGACC	GTAGTCCATTTGTTGGAGAGCGTAAACTATCTCTCC
cLUC-SnRK2.6	TCCCGGGGCGGTACCATGGATCGACCAGCAGTGAGT	GTAGTCCATTTGTTGCATTGCGTACACAATCTCTCC
cLUC-SPL9-N	TCCCGGGGCGGTACCATGGAGATGGGTTCCAACT	TGTAGTCCATTTGTTGGTAACGAGAAGCTAACACAGAG
cLUC-SPL9-C	GTCCCGGGGCGGTACCATGGGGAGGATCGCACCTTCGCT	GTAGTCCATTTGTTGGAGAGACCAGTTGGTATGG
cLUC-ABI5-N	GTCCCGGGGCGGTACCATGGTAACTAGAGAAACGAA	TGTAGTCCATTTGTTGATGTTCTCTAACCACACCAGCC
cLUC-ABI5-M	GTCCCGGGGCGGTACCATGCCCACTAATCCTAAACCTAA	TGTAGTCCATTTGTTGACCATCCACTACTCTTTTCCTT
cLUC-ABI5-C	GTCCCGGGGCGGTACCATGCCAGTGGAGAAAGTAGTGGA	TGTAGTCCATTTGTTGGAGTGGACAACTCGGGTTCC
ABI5-GST	TGGATCCCCGGAATTCATGGTAACTAGAGAAACGAAGTT	GGCCGCTCGAGTCGACTTAGAGTGGACAACTCGGGTTCC
MBP-SPL9	TTCAGAATTCGGATCCATGGAGATGGGTTCCAACT	TTGCCTGCAGGTCGACTCAGAGAGCCCAGTTGGTATGGT
MBP-SPL3	TTCAGAATTCGGATCCATGAGTATGAGAAGAAGC	TTGCCTGCAGGTCGACGTCAGTTGTGCTTTTCCGCCTT
Em1$_{pro}$-LUC	CTTTTAGATAGATGCATTCACAA	TTTTTGAAGAAAAAAACACG
Em6$_{pro}$-LUC	TGGGATGGTACTATATTGGTG	GGCTCTTTAGTTACTTACACAA
SPL9$_{pro}$-GFP	ATTACGAATTCGAGCTCATTTAATACTACTTAAATTTAA	CCGGACTTAAGACTAGTGTTGGTTTCCTCTTACTCAGAC
SPL9$_{pro}$-rSPL9-GFP	ACGAGCTGTACAGATCTATGGAGATGGGTTCCAACTCGG	CGGCCGCTTTAAGATCTTCAGAGAGACCAGTTGGTATGG
SnRK2.6-Flag	CACGGGGGACTCTAGAATGGATCGACCAGCAGTGAG	CCTTGTAGTCCATGTCGACCATTGCGTACACAATCTCTC
SPL9-S203A	CAGTGGCGTCACCGTCATGGCAGAT	TGACGCCACTGGCCGCCTCATCAC
SPL9-S281A	AGCTTCTGCAGGTTTTGGCCCGATG	CCTGCAGAAGCTCGCCATGTATTG

Supplementary Table 2 | Constructs used in this study

Construct name	Vector	Description
nLUC-SPL9	p1300-35S-nLUC	LCI
cLUC-SPL9	p1300-35S-cLUC	LCI
nLUC-SPL3	p1300-35S-nLUC	LCI
cLUC-ABI5	p1300-35S-cLUC	LCI
nLUC-ABI5	p1300-36S-nLUC	LCI
nLUC-ABI3	p1300-37S-nLUC	LCI
nLUC-ABI4	p1300-38S-nLUC	LCI
nLUC-SnRK2.2	p1300-39S-nLUC	LCI
nLUC-SnRK2.3	p1300-39S-nLUC	LCI
nLUC-SnRK2.6	p1300-39S-nLUC	LCI
cLUC-SnRK2.2	p1300-39S-cLUC	LCI
cLUC-SnRK2.3	p1300-39S-cLUC	LCI
cLUC-SnRK2.6	p1300-39S-cLUC	LCI
cLUC-SPL9-N	p1300-35S-cLUC	LCI
cLUC-SPL9-C	p1300-35S-cLUC	LCI
cLUC-ABI5-N	p1300-35S-cLUC	LCI
cLUC-ABI5-M	p1300-35S-cLUC	LCI
cLUC-ABI5-C	p1300-35S-cLUC	LCI
ABI5-GST	pGEX4T-1	Pull down
MBP-SPL9	pMAL-c2X	Pull down
MBP-SPL3	pMAL-c2X	Pull down
$Em1_{pro}$-LUC	pGWB35	Transcriptional activity assay in *Nicotiana*
$Em6_{pro}$-LUC	pGWB35	Transcriptional activity assay in *Nicotiana*
35S:rSPL9-Myc	pGWB17	Transcriptional activity assay in *Nicotiana*
nYFP-ABI5	pEarleygate201-YN	BiFC
cYFP-SPL9	pEarleygate202-YN	BiFC
cYFP-SPL3	pEarleygate202-YN	BiFC
SnRK2.6-His	pCOLD	*In vitro* phosphorylation and semi-*in vivo* pull-down
$SPL9_{pro}$-GFP-rSPL9	p1305-35S-GFP	Transgenic plant
$SPL9_{pro}$-GFP-rSPL9(2A)	p1305-35S-GFP	Transgenic plant
SnRK2.6-Flag	p1300-35S-Flag	Transgenic plant

Supplementary Table 3 | Primers used for qRT-PCR

**Primer name**	**Primer sequence (5'-3')**
Em1-Q-F	TTCCTCGCCTCTCCTCCTTTGTGT
Em1-Q-R	TTCCTCGCCTCTCCTCCTTTGTGT
Em6-Q-F	TTCCTCGCCTCTCCTCCTTTGTGT
Em6-Q-R	TCTTGGTCCTGAATTTGGATTCGT
SPL9-Q-F	CAAGGTTCAGTTGGTGGAGGA
SPL9-Q-R	ATGATGAGTAGGACTGGCAGGTG
ACT7-Q-F	TCCATGAAACAACTTACAACTCCATCA
ACT7-Q-R	TGAAGAAGCTCGCCATGTATTG

Supplementary Table 4 | Primers used for ChIP-qPCR assays

**Primer name**	**Primer sequence (5'-3')**
$Em1_{pro}$-A-F	ATTTATCCGAAATATTGCACTTAGA
$Em1_{pro}$-A-R	TTAATCTTAATCCATTTTCTGTTTC
$Em1_{pro}$-B-F	CTAATCCACACCAGATTCTAACATT
$Em1_{pro}$-B-R	TTAGGTCTTTTTCAAGTTTTGAGTT
$Em6_{pro}$-A-F	GGCAACACACAAAATAATTGC
$Em6_{pro}$-A-R	TACGAAGAAGACTATAGTAGCTCGC
$Em6_{pro}$-B-F	AATCAATTTGATCGGAACTGAAC
$Em6_{pro}$-B-R	TGAAAGTAAATATGAATACCTGAG
$Em6_{pro}$-C-F	TAGCATTTGAATCTTTTCAAAGTTG
$Em6_{pro}$-C-R	GGCATCGACTTTAAGCAGATT
$Em6_{pro}$-D-F	AGGAACACAAATAACTTGCCTCGTA
$Em6_{pro}$-D-R	TCTCAATACTACGTGACTACGTCAA
$Em6_{pro}$-E-F	ACGTCATAATTAGAAAAAGTCAAAA
$Em6_{pro}$-E-R	ATTACATACACACTTCACACTTGC
$ACT7_{pro}$-F	CGTTTCGCTTTCCTTAGTGTTAGCT
$ACT7_{pro}$-R	CACAACGCATGCTAAACAGATCTAG
